# Professional Patents

**Published:** 1888-08

**Authors:** W. Storer How

**Affiliations:** Philadelphia, Pa.


AMERICAN JOURNAL

—OF—

DENTAL SCIENCE.

VOL. XXII. THIRD SERIES—AUGUST, 1888. No. 4.

ARTICLE I.

### PROFESSIONAL PATENTS.

BY W. STORER HOW, D. D. S., PHILADELPHIA, PA.

[Read before the Mississippi Valley Dental Association at Cincinnati, Ohio, March, 7, 1888.]

The discussion of any subject is usually clarified by a definition of the title or terms by which it is designated. In the United States of America a patent is an official document which imports that the patentee is the legal owner of the property therein described, and the inventor is thereby legally constituted for a specified term of years the absolute owner of that property, subject indeed to any claimant who can prove himself to be the original and *first* inventor of the property aforesaid. Contingent upon this risk, or "bar," as it is termed, and the further personal bar of abandonment by neglecting to make application for a patent within two years from the public use of the invention, the right of an inventor to his patented property is the clearest and most unassailable right to property on earth, and is so maintainable in law and equity through the highest courts of this country. It is therefore important to keep in mind these two fundamental facts: First; that a new and useful invention *originates* in the inventor; second, that the invention is *property*, the legal title to which is originally vested in the inventor.

The term dental patents is applied to such patents as relate to inventions connected with the *practice* of dentistry. It is important to notice that these inventions are not designed for the use of the laity, but are for use as improvements in practice by members of the dental profession, and now that dentists are formally included in the general designation of members of the medical profession, it is pertinent to our purpose to consider the subject of professional patents in general. The distinction just made is therefore of consequence as limiting the discussion to inventions intended for professional uses; leaving in abeyance without prejudice the subject of devices designed for use directly by the laity.

It has been commonly assumed by medical men that the primary purpose and function of a member of the medical profession should be the relief of such suffering, or diseased, or disabled members of the human family as should have need of and require his professional services. This assumption might have been permissible in part in the days when the priests were also physicians by the assumed right of a divine calling, and this, as well as many other subjects, is greatly befogged through such antique assumptions of authority, etc., by divine right. Modern thought and action are even now adverse to all such assumptions, and the primary principle is becoming recognized that every man is a king under God, and that no man has, or can rightly have, other than humanly delegated authority over his fellow-man. Government by consent of the governed, is the only just rule for the ruler and the ruled. Professional pretensions to peculiar privileges, practices and prerogatives by virtue of an assumed superiority of vocation, that places its laborers above any pecuniary compensation commensurate with the real value of their services, form the fundamental fallacies which underlie the delusions so prevalent in the minds of both professional men and laymen on the subject of professional patents. It is certainly not true that the professional services of the physician, or surgeon, or dentist are beyond price because they are preservative of human life; for so also are the services of the food-raiser, because food is essential to the preservation of human life,—*ergo*, food is of inestimable value! Now is it true of the doctor any more than of the farmer that he works without expectation of payment or hard cash for his work. Such assumptions are the veriest stuff and nonsense; for whenever and wherever men of any calling work for a living they work for pay,—honorable pay, if the work is honestly done. On the other hand, men who do not work for a living are either gratuitous laborers or lawless loafers, and to this latter class belong the whole group of pretenders from kings to beggars.

Gratuitous service of any kind, under any circumstances, implies the possession of superior abilities by the servant, and a corresponding inability on the part of the served, or, the willingness of the latter to receive service which he is himself able to perform. The inherent degradation of this last disposition it is difficult adequately to describe; and to this subtle debasement of the real manhood of man is owing the inability of the present generation to raise to the true nobility of manliness that must always, in all times and all places, refuse to receive something for nothing if one be able to make any return. Helplessness is, of course, a bar to all self-preservative action, and a man is then simply compelled to receive what is given. A work of love is always and only rewardable by love, and we are not unmindful of the Christian faith that works by love as the highest form of a divinely inspired activity. But the present considerations are based upon and strictly relative to the working fact that men are not yet generally doing business on pentecostal principles, and until the legal fences are all down and the debris cleared away,—the law having vacated its tutorship after bringing all men to Christ,—we should aim to do all things lawfully, with strict regard to all the rights of our neighbors.

Among these rights we have shown that patent rights are of the first order as initiating clear titles to property in inventions. We have also shown that when the inventor is a professional man his property may be of value to other professional men, who may buy it to aid them in their professional work. It has furthermore been seen that professional men properly and primarily work for a living,—*i. e.*, for money in return for services,—as it is eminently right and fit that they should do. The claim that professional services are essentially different from other services for pay is erroneous, as has likewise been shown.

A clear conclusion is, therefore, that the professional man has no more right to the free use of the patented property of his fellow-practitioner than to his other property,—as, for instance, his house, or his horse, or his case of instruments. He might in an emergency borrow anything that is his neighbor's, but to claim the right to use in the practice of his profession a professional invention, on the ground that individual professional property is, *per se*, common professional property, is, assuredly, an untenable proposition. Equally so is the commonly received doctrine that it is unprofessional to patent inventions designed for professional use.

To secure a copyright on a book which is designed for professional use is not now deemed unprofessional, as indeed it ought not to be; but will any right minded person pretend that copyright property is essentially different from patent-right property? They are certainly identical so far as they are original creations of inventive minds, and as such, when secured to the inventive authors by due process of law, are indubitably *property* in the highest and strongest and clearest titular significance of that term. It follows, therefore, that a book, an instrument, or an appliance originated and designed for use by a physician, surgeon, or dentist *professionally* may be copyrighted or patented by its inventor without in any manner or degree derogating from his standing as a professional man. The instrument, as well as the book, is intended to facilitate professional practice, and there is no valid reason why the professional inventor should *give* his invention to his fellow-practitioners any more than that the professional writer should send every fellow a copy of his book with the "compliments of the author." If the book is designed and adapted to promote professional practice, and so alleviate suffering and prolong life, then, on every premise and by every argument used in opposition to professional patents, the book belongs to the profession, and to copyright it or publish it for the personal profit of the author is,—well, all the delusive and deceitful and debasing drivel that has been poured out on inventors by copy-righting authors, and other inconsiderate or pecuniarily interested members of the medical profession. Possibly some introspection is necessary to disclose to many minds the motives which really actuate them when they raise such an outcry against professional patents. They want the best instrument or medicament obtainable, and they want such at the least cost, or without cost if possible. Success in practice depends upon their superiority of repute as professional men. Hence they want the best aids to practice within their reach; and if those aids are only to be had by paying a sum, a part of which goes into the pocket of an inventive fellow-practitioner, a part to the retail dealer, a part to the wholesale dealer, and a part to the manufacturer (or publisher,) then they have a grievance against the inventor, who, as a professional brother (?), ought to give away his part of the profits arising from the sale of his own original property! Why? Well,—because the invention enables the practitioner to do more for suffering humanity, and get more money from suffering humanity, than he could do or get without the invention! The inventor may get any amount of honor (?), but must get no cash for his invention, which belongs to everybody else because the inventor is a professional man; and in the professions, every brother practitioner has the right to take for his own professional use and profit anything which his brother has, or can invent, that is worth taking!

The notion that a professional man has gratuitously received his equipment, and therefore is in honor bound to give all he has and knows to his professional brethren, has no foundation in fact; for the office tuition, the copyrighted books, the college expenses, etc., at every point of his preliminary progress have cost him cash (or its equivalent), which in great measure went into the pockets of the most prominent professors, who are the loudest in professing the greatest concern for the protection of the profession from dishonor, by the patenting of professional inventions. The fact is patent that every medical or dental graduate has been called upon for cash in payment for the professional instruction he has received, and he in turn expects to receive cash for his outlay, just as any other business man expects a return for his investments in knowledge or the products of knowledge.

In the light of to-day, and in the United States of America, it is absurd to assume that the professions are other than departments of business, and distinguishable simply by name from the departments of agriculture, mechanics, merchantry, banking, etc. The other assumption, of the priceless value of medical knowledge as a ground of superiority over the priceless knowledge of the farmer, we have seen to be baseless.

An incidental demonstration of the fallacy of these assumptions has been wrought out in the practice of dentistry, which has by a process of synthesis started from the basis of a business, and by the application of business methods worked its way into the medical profession.

It seems capable of demonstration that the great advances made by modern dentistry in the United States are largely due to the patented inventions of dentists. The writer is free to admit that his own slight participation in this line of progress may unwittingly warp his judgment in this regard, but he is nevertheless firm in his conviction that the opinion is sustained by the facts. In this connection it appears proper to be still further personal in an allusion to the writer's later opportunities for becoming conversant with some correlative phases of the subject.

It is well known to members of this society that the writer was a practitioner of dentistry in this city for nearly thirty years,—less the interval of the war period. The last five years have been passed in Philadelphia, in professional connection with the principal dental manufacturing company of this country,—or, he may add, of the world. Opportunity has been thus afforded for the consideration of dental patents in many aspects unfamiliar to the ordinary practitioner; indeed, the services rendered by the writer have been frequently in the line of a patent expert for the determination of questions concerning inventions of that class. Under those circumstances, and under the present circumstances, when surrounded by old friends and neighbors, a freedom of expression is permissible that might otherwise be deemed inappropriate. Your essayist, therefore, feels at liberty to say that, in five years of confidential and intimate relationship with the heads of the great manufacturing house referred to, he has seen in the conduct of their business not only nothing to condemn, but contrariwise, the most commendable consideration of the interests of dentists, dealers in dental goods, clerks, salesmen, workmen, workwomen and employees of every kind; combined with a general business conduct based upon the highest principles of commercial honor and probity. There need be no hesitation in the use of superlatives when speaking of the sterling qualities of the men who control this great concern. They reflect honor upon the profession which they

—following in the footsteps of the revered founder of the house which bears his name—have sedulously sought to promote, and have been largely instrumental in advancing to its present proud position among the noble and learned professions. Doubtless it is due to the writer's experience in the service of this company that he has reached the firm conclusion that honorable motives and conduct are not confined within the limits of the so-called honorable profession, but that the merchants, the mechanics, and the workmen of every class and degree are honorable, and to be honored whenever and wherever they acquit themselves like true and faithful men.

It is furthermore to be said that the president of the White house, as editor of the *Dental Cosmos*, has rendered inestimable service to dentists and the dental profession during his connection with and conduct of that journal from its beginning, thirty years ago. What is implied in the personal supervision of three hundred and sixty monthly issues of a journal whose every page was to be and has been resolutely kept free of matter not related to dentistry, nor expressed in good, clean English, no one can know but the inflexibly faithful and thoroughly competent editor. What the profession and the world knows is that the *Dental Cosmos* is an honor to dentistry, to journalism, and to the professional business men who have made and kept it foremost and highest.

The elevating nature of dental patents was primarily recognized by this house, and large sums were spent in acquiring and developing them. Few persons are aware of the rapidity with which inventions have followed one another under the stimulus of a reasonable expectation of reward for such extraordinary exercise of thought and skill. Yet fewer persons know what a costly thing it is for the manufacturer to change or discard devices which, when just ready for the market, become improved, or superseded, so that the new stock is rendered comparatively valueless. Just at this point the nerve, and sagacity, and determination of this company to put forth the *very best* goods, that could be made, have time after time been made manifest, and thousands of dollars' worth of superseded things have been destroyed, or shelved without the slightest hesitation, or attempt to realize by holding back the new until the old have been sold. The "monopoly" against which such thoughtless outcries have been made is of untold benefit to dentists because both able and willing to discard the old good things immediately upon the advent of the new and better ones. So, too, have the foremost dentists always done, and that is the secret of success,—the best, at whatever cost. That is also at once the inspiration and hope of the inventor.

To summarize our conclusions, it may be confidently declared that:

*First.*—An original and first inventor has ***absolutely produced*** the ***property*** to which he is entitled by his patent.

*Second.*—It is strictly right, equitable, ethical, just and proper for an inventor who is either a clergyman, lawyer, physician, surgeon, dentist, chemist, author, composer, or worker in any of the departments of business termed professions, to patent his invention, whatever its object, if, and if only, it be useful to such of his fellowmen as may properly desire to use it lawfully.

*Third.*—The outcry against professional men who do, or countenance those who do, obtain patents for human life-saving or life-promoting inventions comes in every instance from men who either do not understand the subject, or are directly or remotely interested in obtaining the use of the invention gratuitously, or without due process of law.

As products of thought, the movable types, the cotton gin, the steam engine, and the electro-magnetic telegraph have been directly and indirectly instrumental in saving and promoting life in possibly far greater degree than all the medical prescriptions of the contemporaneous physicians whose diplomas invested them with exclusive rights and privileges as a means of making money; yet these same professional diplomatists would proscribe a fellow-prescriber if he were to obtain a Patent Office diploma as the originator of a life-saving invention.

If, therefore, common sense, reason, argument, logic, demonstration, humanity, beneficence, lawfulness and righteousness are not all on the side of the professional inventor and patentee, then his case only needs restatement in embossed letters that may enable the wilfully blind to mentally see the affirmative facts that are indubitably clear to the mind of an unprejudiced investigator. The great number of persons to whom such an invention should prove a priceless possession might be a stimulus to productive ingenuity, were it not for the amazing probability that nearly every one of them would bring forward either his professional brotherhood or his great need of the professional equipment as an unanswerable plea for the gratuitous bestowment of the property upon himself; the *honor* of being the inventor of so useful a device sufficing (of course) as an ample reward for the inventor in lieu of the filthy lucre fees which would be what the practicing professor would obtain from its use.

The phrase "empty honors" is certainly a significant one, and must have been devised for a professional inventor, because he, of all other men, would have been, as in modern times, sure to have received neither cash nor substantial gratitude, however valuable his invention might have proved to his fee-earning brethren. History is full of examples uniformly tending to show that, if the inventor could not *compel* the recognition of his property rights in his invention, he has not only been denied compensation, but nas often suffered persecution, and even martyrdom.

In these later years, when every other department of practical knowledge and labor is making such progress by means of patented inventions that the Old world stands amazed and almost awe struck at sight of the marvels wrought in the New, is it not time for the old professions to fall into line with the new, and declare that henceforward every incentive shall be held out to the ingenious and skillful professional man to invent or discover human life-saving and health-promoting means and devices, with the assurance that his patent rights shall be respected, and due honors be paid with the cash for the privilege of professionally using his invention?

Will it not be an ascent to a higher plane of thought and action for "doctors" (teachers) to teach that professional honor is not a cloudy abstraction, nor a clannish distinction, nor an exclusive assumption; and that, in particular, a doctor of medicine or surgery is a person called to and trained in the professional business of the readjustment and repair of disordered human bodies for a cash consideration, due and to be paid upon the completion of the service?

If it be objected that this view degrades the practice of a profession to the merely mercantile prosecution of a business, it is at once to be said that the objector begs the question by the false assumption that a business is beneath a profession, whereas an honest business honorably conducted puts the business man above beggary of every kind. He never begs, but buys and sells with the most scrupulous regard to the property rights of everybody; and, to his great credit, it is to be said that there is among men no higher working standard of honor than that of the business man whose simple statement or working formula is "I will sell this to-day for so much," and when his fellow-merchant at hand, or a thousand miles away, replies, "I will take it at your price," that practically completes the transaction, and the property will be transferred, although only their words of honor will have passed between them, and although, also, prior to the transfer of the property in fact, its value should change to the amount of many thousands of dollars; the business man's word is actually as good as his bond.

There is, therefore, relevancy to the subject under consideration in the writer's illustrative allusion to the wellknown business house in which he has learned many instructive things concerning the correlations of professional honor with business honor, and as common rules of practice, the terms appear to him to be not as yet synonymous.

As a matter of fact, it is proper to state here that, aside from the young lady type-writer, no person either nearly or remotely connected with the business house referred to is aware of even the intention of the author to write or read a paper upon this subject; and, therefore, the responsibility for the views and sentiments herein presented rests entirely and exclusively upon him.

---

As supplemental to the foregoing paper, the author adds to this publication of it on his own account, for personal distribution, the following extract, in order that the reader may verify the irrefutable fact that the exclusive copyright and the exclusive patent right are fundamentally identical in their establishment under the law which was intended "To promote,"—and has to a marvelous degree practically promoted,—"the progress of science and useful arts."

So far as the author is aware, the copyright and the patent right are the only instances of the constitutional creation of property in materialized or embodied thought.

Article I, Section 8, in the Constitution of the United States declares: "The Congress shall have power" . . . "8. To promote the progress of science and useful arts, by securing for limited times to authors and inventors the exclusive right to their respective writings and discoveries." Also, "18. To make all laws which shall be necessary and proper for carrying into execution the foregoing powers, and all other powers vested by this constitution in the government of the United States, or in any department or officer thereof.

